# Testing a Personalized Approach to Chronic Low Back Pain: A Randomized Controlled Trial in Older Veterans

**DOI:** 10.1002/acr.25671

**Published:** 2026-02-08

**Authors:** Debra K. Weiner, Angela Gentili, Meika A. Fang, Edward Garay, Laura Lawson, Lenore Joseph, Cathy C. Lee, Michelle I. Rossi, Beverly Thorn, Subashan Perera

**Affiliations:** ^1^ Geriatric Research, Education and Clinic Center, VA Pittsburgh Healthcare System Pittsburgh Pennsylvania; ^2^ VA Pittsburgh Healthcare System Pittsburgh Pennsylvania; ^3^ Central Virginia VA Health Care System Richmond; ^4^ Virginia Commonwealth University Health System Richmond; ^5^ VA Greater Los Angeles Healthcare System Los Angeles California; ^6^ David Geffen School of Medicine University of California Los Angeles; ^7^ Geriatric Research Education and Clinic Center, VA Greater Los Angeles Healthcare System Los Angeles California; ^8^ School of Medicine University of Pittsburgh Pittsburgh Pennsylvania; ^9^ University of Alabama Tuscaloosa; ^10^ Graduate School of Public Health University of Pittsburgh Pittsburgh Pennsylvania

## Abstract

**Objective:**

We aimed to test the efficacy of personalized treatment of older veterans with chronic low back pain (CLBP) delivered by Aging Back Clinics (ABCs) as compared with usual care (UC).

**Methods:**

Two hundred ninety‐nine veterans aged 65 to 89 with CLBP from three Veterans Affairs (VA) medical centers underwent baseline testing, randomization to ABC or UC, and 12 months of follow‐up. ABC care was guided by trained physicians and published algorithms targeting key conditions contributing to CLBP (eg, hip osteoarthritis, depression, fibromyalgia). UC was guided by the participant's primary care provider. The primary outcome was six‐month change in the Oswestry Disability Index (ODI). Among multiple secondary outcomes were pain intensity, quality of life (Patient‐Reported Outcomes Measurement Information System [PROMIS]‐Global Health), self‐reported physical function (PROMIS‐29), falls, life space, and health care utilization collected at 3, 6, 9, and 12 months. Analyses were conducted according to intention‐to‐treat.

**Results:**

There were no significant group differences in ODI change. Greater improvement with ABC in the PROMIS‐29 physical function scale was observed at 12 months (1.7 vs −0.4 points), PROMIS Global physical health at 6 (1.3 vs −1.2) and 12 months (0.7 vs −1.5), PROMIS Global mental health at 6 months (0.2 vs −2.3), and present and prior week average/worst pain over 12 months (all *P* < 0.05). There were marginally fewer falls over 12 months (*P* = 0.0527).

**Conclusion:**

We did not find confirmatory evidence that personalized care (ABC) was superior with respect to ODI. We did find preliminary evidence that ABC was superior in other respects including improved self‐reported physical health, less pain, and fewer falls.

## INTRODUCTION

Back and other musculoskeletal pains are the most common reasons that United States veterans seek medical care,^1^ and approximately half of such patients are aged 65 and older.^2^ The prevalence of low back pain in those 85 and older, the most vulnerable and fastest growing segment of society, is estimated at 44%.^3^ Chronic low back pain (CLBP; ie, occurring on at least half the days for at least six months^4^) is associated with the overwhelming majority of low back pain–associated health care resource utilization and personal suffering, including physical disability, depression, anxiety, insomnia, cognitive dysfunction, loss of sleep and appetite, and social isolation.^5^, ^6^, ^7^ In older veterans (ie, aged ≥65), physical and emotional suffering may be compounded by a heightened risk of iatrogenic adversity associated with commonly employed interventions such as spinal injections, surgery, and potentially harmful medications (eg, gastrointestinal bleeding and renal failure with nonsteroidal anti‐inflammatory drugs, falls and hip fractures with opioids^8^). Furthermore, although substantial resources continue to target treating patients with back pain, treatment outcomes are typically modest and have remained stagnant.^9^ What accounts for this health care crisis?

**SIGNIFICANCE & INNOVATIONS**

- This is the first clinical trial testing the efficacy of a personalized approach to managing chronic low back pain (CLBP) in older adults, guided by published algorithms that operationalize the evaluation and treatment of conditions that contribute to pain and disability and reside outside the lumbar spine.
- Although we did not find confirmatory evidence of reducing self‐reported CLBP disability, we did find some evidence supporting reduction of pain intensity and falls as well as improvement in self‐reported physical health in participants who received personalized as compared with usual care.

CLBP is referred to in the medical literature as a “nonspecific” condition evaluated and managed using imaging‐guided spine‐specific approaches (eg, spinal injections) or generic treatments (eg, pain medications, physical therapy, cognitive behavioral therapy). Spine‐specific approaches may lead to suboptimal outcomes in older adults because imaging‐identified pathology such as degenerative disc disease and spinal stenosis are common in older individuals, even those who are pain free^10^, ^11^; thus, such approaches may or may not target actual contributors to pain. Generically applied first‐line treatments such as physical therapy, cognitive behavioral therapy, and/or oral analgesics typically result in only modest improvement in pain and function,^12^ perhaps not surprising because CLBP is a biopsychosocial syndrome in which disability may be caused by a combination of physical and psychosocial factors for which optimal treatment requires a comprehensive approach.^13^ In older adults, there may be a combination of specific CLBP‐associated disability contributors such as hip osteoarthritis, myofascial pain, fibromyalgia, and depression that cannot be detected by spinal imaging but for which evidence‐based treatments exist.

The present manuscript reports the results of a clinical trial based on the premise that CLBP in older adults is a geriatric syndrome, that is, a final common pathway for the expression of multiple specific contributors.^14^ The lumbar spine is considered the weakest link (ie, the most vulnerable part of the system), and multiple conditions outside it are considered potential treatment targets.^15^ We have published a series of evidence and expert opinion‐based clinical evaluation and treatment algorithms for key conditions that are commonly overlooked or misdiagnosed in older adults with CLBP (ie, hip osteoarthritis, fibromyalgia, myofascial pain, depression, anxiety, insomnia, maladaptive coping, sacroiliac joint syndrome, leg length inequality, lateral hip and thigh pain, lumbar spinal stenosis, dementia)^16^, ^17^, ^18^, ^19^, ^20^, ^21^, ^22^, ^23^, ^24^; we have explored their prevalence in older veterans^25^ and conducted a pilot study demonstrating feasibility and preliminary efficacy of algorithm‐guided care.^26^ We hypothesized that older veterans with CLBP who are randomized to receive care in Aging Back Clinics (ABCs) that conduct evaluations and prescribe treatment using a comprehensive, algorithm‐guided approach will have greater 6‐month (primary endpoint) and 12‐month reductions in pain‐related disability (primary outcome) and improvements in other outcomes as compared with those who receive usual care (UC).

## PARTICIPANTS AND METHODS

### Study participants

The parent study methodology is described in detail elsewhere.^27^ The study was approved by the Veterans Affairs (VA) Central Institutional Review Board (no. 18‐23, approved 9‐13‐2018). Briefly, 299 veterans aged 65 to 89 with CLBP, on average, of at least moderate severity (based on a verbal descriptor scale) were recruited from three VA sites (VA Pittsburgh Healthcare System, Pittsburgh, Pennsylvania; Central Virginia VA Health Care System, Richmond, Virginia; West LA VA Medical Center, Los Angeles, California). Participants were excluded if they did not speak English or had other communication difficulties (severe uncorrected vision or hearing loss), screened positive for possible dementia based on a Folstein mini‐mental state examination (MMSE)^28^ score less than 24 of 30 (because the psychometric properties of our outcome measures have not been established in those with dementia), had psychotic symptoms, were acutely ill, had previously undergone lumbar surgery, had pain in other body locations more severe than that in the lower back, reported active illicit substance use, or had “red flags” (ie, signs or symptoms indicative of serious underlying illness requiring urgent care for their low back pain). Race and ethnicity were obtained from the participant's electronic medical record.

### Testing procedures

#### Baseline

The following parameters were assessed on all participants at baseline: (1) the Oswestry Disability Index (ODI)^29^, our primary outcome measure, assesses interference of pain with function; (2) the Minimal Data Set (MDS), recommended by the National Institutes of Health Task Force on research standards for CLBP,^4^ measures pain severity and interference with daily activities, widespread pain, prior CLBP treatments, overall physical function, depressive symptoms, sleep, psychological maladaptation (ie, fear‐avoidance beliefs and catastrophizing), alcohol/drug use, cigarette smoking, demographics (age, race, ethnicity, gender, education, marital status), height, and weight; (3) the Patient‐Reported Outcomes Measurement Information System (PROMIS)‐29 items not already included in the MDS—anxiety symptoms, fatigue, and participation in social roles and activities^30^; (4) other key cofactors that could contribute to disability—medical comorbidity (Duke comorbidity index^31^), pain medications (opioids and nonopioids), social support (Medical Outcomes Study Social Support Scale^32^), opioid difficulties in those taking opioids (Prescribed Opioids Difficulties Scale^33^) and smoking status; (5) pain severity rated 0 to 10 for pain now, average pain over the past week, and worst pain over the past week; (6) the Pain Self‐Efficacy Questionnaire^34^ assesses the confidence people with ongoing pain have in performing activities while in pain; (7) quality of life was assessed with (a) the PROMIS Global Health scale and (b) the Veterans RAND 12 Item Health Survey (VR‐12), a measure of health‐related quality of life comprised of 12 items and collapsed into two summary variables—physical health and mental health; (8) the Quick Mild Cognitive Impairment (QMCI) screen is a validated measure that screens for the presence of mild cognitive impairment^35^ (participants with more significant cognitive impairment were already excluded using the MMSE); (9) balance confidence was evaluated with the Falls Efficacy Scale‐international short form^36^; (10) falls were assessed as the number of self‐reported falls over the prior three months; and (11) mobility was assessed with gait speed and life space, the spatial extent of a person's mobility.^37^

#### Intervention phase

Procedures conducted during the intervention phase are described in detail elsewhere.^27^ Following collection of baseline measures, participants were randomized in equal proportions to receive either UC or algorithm‐guided personalized CLBP care in ABCs stratified by VA site and using random block sizes of four and six (chosen with equal likelihood). Those randomized to UC were instructed to discuss their CLBP care with their primary care provider. Those randomized to ABC care received a personalized treatment regimen by ABC providers.

ABC providers were VA medical center physicians. At the Pittsburgh VA, there were three providers—two geriatricians and one physiatrist/pain medicine physician. At the Richmond VA, there were three providers—two geriatricians and one neurologist. At the West LA VA there were two providers—one geriatrician and one rheumatologist.

ABC providers received structured training from the principal investigator (PI) on the application of our published algorithms on diagnostic criteria and management guidelines of 11 separate conditions contributing to CLBP. The PI also assessed treatment implementation fidelity (ie, adherence to treatment algorithms) by conducting a monthly review of structured research notes and providing ABC providers with written feedback.

At the initial ABC visits, the study coordinators asked the veterans to complete a self‐report tool on an electronic tablet, *Take Back Your Back*, to screen for conditions contributing to their CLBP and related disability (eg, hip osteoarthritis, depression, fibromyalgia) and help guide the clinical assesment.^38^ The ABC providers reviewed the results of the screening tool and conducted a structured history and physical examination to identify the specific treatment targets. As an example, if the participant screened positive for depression on the Patient Health Questionnaire‐2, they would conduct a more detailed assessment for depression. ABC providers educated veterans on the clinical findings and the conditions contributing to the CLBP and then collaborated with veterans to design a multifaceted personalized care plan with specific treatment recommendations for each condition as guided by our algorithms. An educational booklet was provided to patients detailing their personalized treatment targets and plans.

As the management plan was based on a collaborative approach, veterans were free to choose some and decline other treatment recommendations. The ABC providers referred participants to the appropriate services (eg, physical therapy, chiropractic, acupuncture, behavioral health) and conducted follow‐up visits as they felt appropriate. The number of treatments was determined by the providers to which veterans were referred. Veterans were permitted to continue identified treatments for the duration of the follow‐up period or as long as deemed appropriate by the receiving service. If acute issues arose before their scheduled follow‐up appointments with ABC providers, veterans were permitted to reach out to schedule an earlier appointment.

#### Follow‐up

In the ABC group, the ABC provider scheduled follow‐up visits as clinically indicated, with the last visit scheduled at the end of the study. The primary outcome point was at six months following randomization. The follow‐up period lasted up to 12 months. In both the ABC and UC groups, measures were collected over the telephone by a research coordinator masked to randomization assignment, either monthly (pain intensity and health care utilization), every 3 months (ODI, PROMIS‐29, falls and falls efficacy, PROMIS global health, VR‐12), or at 12 months (medications, global impression of change). Health care utilization was assessed as numbers of office visits, emergency room visits, hospitalizations, opioid prescriptions, and back injections in both intervention arms. At the final study visit, participants were asked to evaluate the perceived value of the various treatments that they received, from 0 = not helpful to 10 = extremely helpful.

**Figure 1:**
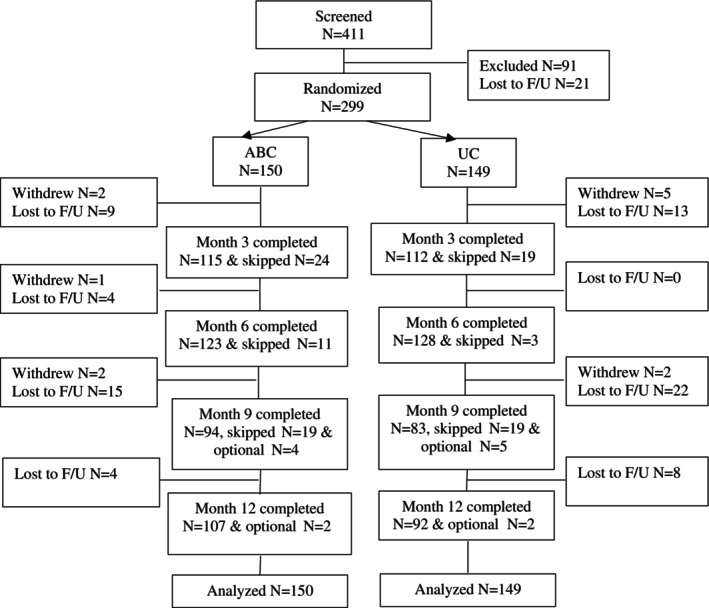
Study participant flow. ABC, Aging Back Clinic; F/U, follow‐up; UC, usual care.

For the 9‐month and 12‐month data collection time points, data were collected on a subset of participants. The clinical trial was conducted during the peak of the COVID‐19 pandemic and recruitment was often challenging, with recruitment extending beyond the planned period. The final participants in the trial were asked to volunteer to be observed for only six months, as it was the primary study end point. This is reflected in Figure 1, which annotates some participants as “optional.”

### Statistical analysis

Prior data and assumptions include a between‐subject SD of 18 points for pre‐ to postintervention change in the primary outcome ODI^39^ and a conservative dropout rate of 15% from other back pain trials.^40^ With 330 participants randomized and 280 anticipated completers, we sought be able to detect an observed primary outcome difference as small as seven points with 90% power in a two‐tailed test at the α = 0.05 level. Due to the aforementioned challenges due to the COVID‐19 pandemic, we were able to randomize only 299 participants, which was estimated to afford 87% statistical power under the same assumptions.

We used appropriate descriptive statistics to summarize participant characteristics and outcomes by intervention group and time point. Participant baseline characteristics were compared between intervention arms using independent samples *t*‐ and chi‐square tests, as appropriate. The main analyses were conducted on an intention‐to‐treat basis and multiple imputation for missing data in continuous variables from the first six months. Specifically, for continuous outcomes (including the primary outcome), we fitted a series of linear mixed models with change from baseline as the dependent variable; intervention group, follow‐up time point, and their interaction as fixed effects of interest; baseline value of the outcome as a fixed effect covariate; and participant as a random effect. Means contrasts were constructed to estimate the adjusted between‐group difference at each follow‐up time point. For count outcomes representing health care utilization and falls, we fitted negative binomial regression models with logarithm of reporting months as an offset (exposure) and intervention group as the independent variable, and we estimated incident rate ratios to quantify the intervention effects. SAS software version 9.4 (SAS Institute, Inc.) was used for statistical analysis.

## RESULTS

Participant flow is shown in Figure 1. A total of 411 veterans were screened and 299 were randomized. Supplemental Table 1 lists reasons for exclusion. All follow‐up data were collected by telephone. There were no reportable adverse events.

Participant baseline characteristics are displayed in Table 1. Mean age was approximately 73 years, most participants were male, more than two‐thirds were White, approximately a quarter were Black, and majority had attended at least some college. There were no significant between‐group differences, with the exception of a greater percentage of participants in the ABC group reporting refreshing sleep and more participants in the UC group with sleep disturbance and worse QMCI performance on the clock drawing and logical memory tests.

**Table 1:** Participant baseline characteristics*

Participant characteristic	ABC (n = 150)	UC (n = 149)	*P* value^a^
Age	73.5 ± 5.1	73.3 ± 5.0	0.7956
Male gender	136 (90.7)	143 (96.0)	0.0663
Hispanic ethnicity	11 (7.3)	14 (9.4)	0.5194
Race			0.5653
Asian	0 (0.0)	3 (2.0)	
Black	41 (27.3)	41 (27.5)	
Native American	2 (1.3)	2 (1.3)	
Pacific Islander	1 (0.7)	1 (0.7)	
White	105 (70.0)	99 (66.4)	
Unspecified	1 (0.7)	3 (2.0)	
Education			0.4162
High school or less	50 (33.6)	40 (26.7)	
At least some college	75 (50.3)	85 (56.7)	
Graduate/professional degree	24 (16.1)	25 (16.7)	
Oswestry Disability Index	30.5 ± 14.7	33.1 ± 15.3	0.1239
Duke Comorbidity Index	3.4 ± 1.3	3.6 ± 1.3	0.2165
Cardiac	35 (23.3)	33 (22.2)	0.8068
Neurologic	14 (9.3)	22 (14.8)	0.1490
Musculoskeletal	140 (93.3)	136 (91.3)	0.5043
General	90 (60.0)	91 (61.1)	0.8494
Visual/hearing	114 (76.0)	122 (81.9)	0.2126
Diabetes	44 (29.3)	49 (32.9)	0.5070
Cancer	39 (26.0)	43 (28.9)	0.5795
Lung	35 (23.3)	40 (26.9)	0.4836
Minimum data set			
Height, in.	68.5 ± 3.4	69.5 ± 3.3	0.0119
Weight, lb	209.2 ± 42.7	212.4 ± 38.2	0.4930
Body mass index	31.5 ± 6.5	31.0 ± 5.4	0.5009
Average pain	5.6 ± 1.8	5.6 ± 1.9	0.1213
Pain duration 5+ y	118 (78.7)	120 (80.5)	0.6882
Pain every day	133 (88.7)	128 (85.9)	0.4736
Leg pain	55 (36.7)	52 (34.9)	0.7499
Bothersome stomach pain	111 (74.0)	110 (73.8)	0.9726
Bothersome joint pain	33 (22.0)	28 (18.8)	0.4913
Bothersome headache	109 (72.7)	97 (65.1)	0.1576
Bothersome widespread pain	76 (50.7)	79 (53.0)	0.6838
Prior low back pain treatments			
Opioid painkiller use (ever used)	63 (42.0)	57 (38.3)	0.5089
Current opioid use	29 (19.3)	33 (22.2)	0.5484
Injections (ever used)	45 (30.0)	45 (30.2)	0.9697
Exercise therapy (ever used)	98 (65.3)	94 (63.1)	0.6854
Psychological counseling (ever used)	18 (12.0)	19 (12.8)	0.8435
Unemployed 1+ mo	6 (4.0)	11 (7.4)	0.2066
Disability/worker's compensation	10 (6.7)	15 (10.1)	0.2882
Felt worthless (sometimes/often/always)	21 (14.0)	21 (14.1)	0.9813
Felt helpless (sometimes/often/always)	23 (15.3)	22 (14.8)	0.8907
Felt depressed (sometimes/often/always)	29 (19.3)	35 (23.5)	0.3809
Felt hopeless (sometimes/often/always)	12 (8.0)	15 (10.1)	0.5329
Sleep quality (good/very good)	52 (34.7)	50 (33.6)	0.8396
Refreshing sleep (somewhat/quite a bit/very much)	106 (70.7)	86 (57.7)	0.0195
Problems with sleep (somewhat/quite a bit/very much)	83 (55.3)	92 (61.7)	0.2605
Difficulty falling asleep (somewhat/quite a bit/very much)	49 (32.7)	60 (40.3)	0.1721
Unsafe to be physically active (fear‐avoidance belief)	39 (26.0)	43 (28.9)	0.5795
Pain is terrible and never going to get better (catastrophizing)	55 (36.7)	61 (40.9)	0.4484
Drinking/drug use	12 (8.0)	18 (12.1)	0.2403
Need to reduce drinking	11 (7.3)	13 (8.7)	0.6579
Smoking status:			0.5145
Never smoker	48 (32.0)	40 (26.9)	
Current smoker	19 (12.7)	24 (16.1)	
Quit smoking	83 (55.3)	85 (57.1)	
Pain medications	103 (68.7)	100 (67.1)	0.7737
Opioid pain medications	17 (11.3)	14 (9.4)	0.5827
MOS social support			
Emotional/informational	76.3 ± 24.3	75.7 ± 23.5	0.8344
Tangible	78.4 ± 25.6	78.6 ± 24.4	0.9360
Affectionate	75.3 ± 24.9	72.1 ± 26.7	0.2869
Positive social interaction	78.6 ± 24.2	75.5 ± 24.6	0.2808
Overall	77.2 ± 23.7	76.0 ± 23.3	0.6605
Prescribed opioids difficulties scale			
Psychological problems	0.4 ± 2.2	0.7 ± 3.0	0.2415
Opioid control concerns	0.3 ± 1.7	0.5 ± 2.2	0.3333
Overall	0.7 ± 2.8	1.2 ± 4.2	0.1684
Opioids helpful (at least a little)	21 (14.0)	25 (16.8)	0.5055
MMSE	28.6 ± 1.6	28.5 ± 1.6	0.6186
Pain scale			
At the moment	4.0 ± 2.7	4.2 ± 2.6	0.4736
Prior week average	5.7 ± 2.0	6.0 ± 2.0	0.2691
Worst	8.0 ± 1.8	8.1 ± 2.1	0.7005
Pain self‐efficacy scale	44.6 ± 11.6	43.5 ± 12.6	0.4574
PROMIS‐29			
Physical function	39.2 ± 7.2	39.0 ± 7.5	0.8491
Anxiety scale	46.8 ± 8.4	48.1 ± 8.9	0.1716
Depression scale	46.7 ± 7.7	46.9 ± 7.7	0.8679
Fatigue	51.6 ± 8.8	51.1 ± 9.6	0.6559
Sleep disturbances	51.1 ± 7.6	53.0 ± 8.4	0.0419
Social roles	50.1 ± 8.7	48.7 ± 9.8	0.2027
Pain interference	59.0 ± 6.4	59.7 ± 7.6	0.4421
Average pain	5.6 ± 1.8	5.9 ± 1.9	0.2232
PROMIS Global Health			
General health	3.3 ± 0.9	3.0 ± 0.9	0.3561
Roles	3.3 ± 0.9	3.4 ± 1.1	0.7132
Physical health	41.4 ± 6.0	41.6 ± 7.3	0.7220
Mental health	48.8 ± 8.3	49.2 ± 78.7	0.7061
QMCI			
Orientation	10.0 ± 0.3	9.9 ± 0.4	0.6143
Word registration	4.7 ± 0.6	4.6 ± 0.6	0.1109
Clock drawing	13.3 ± 2.4	12.5 ± 2.9	0.0186
Delayed recall	13.1 ± 5.2	12.6 ± 5.3	0.4940
Verbal fluency	14.1 ± 5.0	14.3 ± 5.0	0.7972
Logical memory	19.1 ± 5.3	17.6 ± 5.7	0.0190
Total score	74.2 ± 12.1	71.5 ± 12.5	0.0647
VR‐12			
Physical summary	35.2 ± 10.0	35.2 ± 10.5	0.9613
Mental summary	53.8 ± 10.2	53.6 ± 9.8	0.8579
Gait speed, m/s	0.72 ± 0.25	0.70 ± 0.23	0.3233
Life Space Assessment	69.5 ± 27.3	69.0 ± 27.5	0.8939
Falls Efficacy Scale	12.3 ± 5.5	12.7 ± 5.9	0.5466
Falls prior 3 mo			
Any	40 (26.7)	46 (30.9)	0.4072
Multiple	23 (15.3)	25 (16.8)	0.6953

* Values are in mean ± SD or n (%). Computed using independent samples *t* tests for continuous variables, and chi‐square and Fisher's exact tests for categorical variables. ABC, Aging Back Clinic; MMSE, mini‐mental state examination; MOS, Medical Outcomes Study; PROMIS, Patient‐Reported Outcomes Measurement Information System; QMCI, Quick Mild Cognitive Impairment; UC, usual care; VR‐12, Veterans RAND 12 Item Health Survey.

^a^ Using independent samples *t*‐ or chi‐square tests.

**Figure 2:**
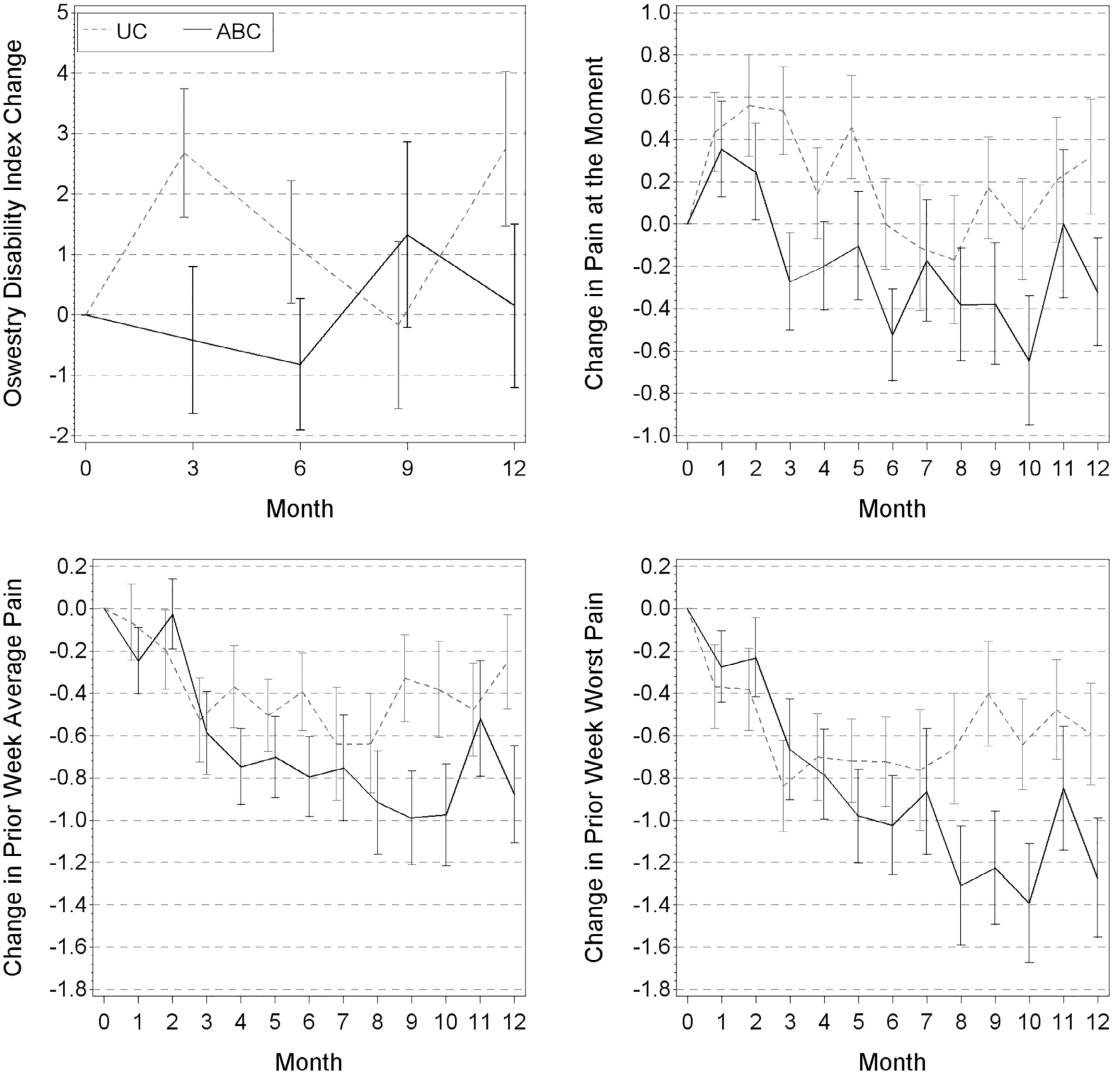
Changes in pain and related disability between usual care (UC) group and aging back clinic (ABC) group.

Table 2 and Figure 2 display change of the ODI, the primary outcome. There were no significant differences in ODI change (the primary outcome) at any of the time points. Significant differences in several of the secondary outcome changes were observed, as displayed in Supplemental Table 2. ABC group showed greater improvement in the PROMIS‐29 physical function scale at 12 months (1.7 vs −0.4 points), PROMIS Global general health at 12 months (−0.2 vs −0.4), PROMIS Global physical health scale at 6 (1.3 vs −1.2) and 12 months (0.7 vs −1.5), PROMIS Global mental health at 6 months (0.2 vs −2.3), and present and prior week average/worst pain over 12 months (all *P* < 0.05; Figure 2).

**Table 2:** Continuous primary outcome changes from baseline by intervention group*

Outcome and period of change, Oswestry Disability Index	ABC (n = 150), mean ± SD	UC (n = 149), mean ± SD	Adjusted difference,^a^ mean ± SE	*P* value
3 mo	−0.4 ± 13.0	2.7 ± 11.3	−2.7 ± 1.6	0.0982
6 mo^b^	−0.8 ± 12.0	1.2 ± 11.5	−2.0 ± 1.5	0.1820
9 mo	1.3 ± 14.7	−0.2 ± 12.6	−0.3 ± 1.7	0.8738
12 mo	0.2 ± 14.0	2.8 ± 12.1	−2.8 ± 1.6	0.0844

* ABC, Aging Back Clinic; UC, usual care.

^a^ Adjusted for preadministration value of the physiologic measure as a covariate using linear mixed models and multiple imputation for missing data.

^b^ Primary outcome and endpoint.

Table 3 displays effect on count outcomes representing health care utilization and falls. There were more office visits in the ABC group (2.3 vs 1.78 per person‐month; *P* = 0.0287) and marginally significantly fewer falls (0.23 vs 0.36 per person‐month; *P* = 0.0527).

**Table 3:** Effect on healthcare utilization outcome (6–12 months) and falls (over 12 months)*

Outcome	ABC, rate per person‐month	UC, rate per person‐month	Incident rate ratio^a^ (95% CI)	*P* value
Office visits	2.30	1.78	1.59 (1.05‐2.41)	0.0287
Emergency room visits	0.08	0.06	1.78 (0.64‐4.95)	0.2658
Hospital visits	0.03	0.02	1.59 (0.52‐4.84)	0.4172
Opioid prescriptions	0.01	0.02	0.74 (0.23‐2.35)	0.6064
Back injections	0.00	0.01	0.24 (0.01‐10.5)	0.4561
Falls	0.23	0.36	0.39 (0.15‐1.01)	0.0527

* ABC, Aging Back Clinic; CI, confidence interval; UC, usual care.

^a^ Estimated using a negative binomial regression model unless otherwise noted.

Supplemental Table 3 describes the conditions identified at baseline by the ABC provider and the services to which participants were triaged for the length of their study participation. Each condition screened was present in at least 20% participants, with the exception of fibromyalgia (present in 2.7%) and leg length discrepancy (present in 11.3%). One or more central nervous system (CNS) conditions (anxiety, depression, fibromyalgia, insomnia, maladaptive coping) were present in 67.3% of participants. One or more physical conditions (hip osteoarthritis, lateral hip/thigh pain, leg length discrepancy, lumbar spinal stenosis, myofascial pain, sacroiliac joint syndrome) was identified in 90% of participants. Both CNS and physical conditions were present in 64.7% of participants. Services most often referred to varied by condition, and overall were behavioral health, physical therapy, hip injection, and acupuncture. Supplemental Table 4 repeats Table 2 and Supplemental Table 2 analyses with ABC group restricted to participants that were highly compliant with treatment recommended by the ABC provider (assessed by the ABC provider at the final study visit), showing a strengthening of the magnitudes of intervention effect.

Supplemental Table 5 displays data on the frequency of various treatments received during the course of the study and participants perceived value of these treatments. For the majority of treatments queried, more participants in the ABC group received treatment than those in the UC group. For shots in the spine/muscle and massage, more participants in the UC group received these treatments than those in the ABC group. Although a similar percentage of participants were taking opioids in the ABC group (19.3%) and the UC group (17.4%), their perceived value was greater in the UC group (mean rating 6.6) as compared with the ABC group (mean rating 5.3). For the acupuncture and two education treatments, their perceived value was more than one point greater in the ABC group (6.3, 4.9, 5.2) than in the UC group (4.4, 3.5, 3.4). For yoga, its perceived value was greater in the UC group (6.8) than in the ABC group (4.5). For each of the other treatments, their perceived value was similar in both groups.

## DISCUSSION

Older veterans with CLBP who were treated using a personalized, algorithm‐guided approach did not show confirmatory evidence of greater improvement in CLBP‐associated disability as compared with those who received UC. Several secondary outcomes including at‐the‐moment and prior week pain and physical health demonstrated statistically significant evidence of greater improvement, and physical health improvement was also clinically significant based on a two‐point change, the lower end of the published range.^41^ Also, the fall rate demonstrated somewhat weaker evidence of greater improvement in the ABC group compared to the UC group.

We did not demonstrate evidence of a greater improvement in our primary outcome, the ODI, in contrast to other clinical trials that have focused on older adults with CLBP. One trial tested the efficacy of 12 weeks versus 36 weeks of chiropractic spinal manipulation and exercise in 182 older adults with back pain for an average of 18 weeks and demonstrated significant reduction in ODI scores in both groups.^42^ A small study (n = 31) conducted in older women with low back pain for at least 3 months of the prior 12 (mean low back pain duration ~8 years) demonstrated efficacy of core stability training with significant improvement in the ODI.^43^ Another small study (n = 20) that compared two types of physical therapy in older adults with a mean low back pain duration of 12 to 16 months also demonstrated significant ODI improvement immediately after an eight‐week intervention.^44^ However, we do also note the preliminary evidence of efficacy with respect to other secondary outcomes and strengthening of the intervention effects in the per‐protocol post hoc analysis as aspects of consistency with prior studies.

The reason for difference between our findings and those of others is likely multifaceted. In each of the above studies, the frequency and content of the interventions were standardized, but ours were not. Although our algorithms recommend a specific triage path for participants with contributing conditions, the content, frequency, and duration of the treatments themselves were left to the discretion of treating clinicians and collaborative discussions with participants with a view toward pragmatism. In addition, participants were referred to different clinicians and therapists with variable skill sets. Participant compliance (eg, with treatment session attendance and/or follow‐through with a home program) also was very likely quite variable.

Approximately 80% of our participants reported back pain duration of at least five years. In two of the three studies referenced above, low back pain duration was significantly shorter, and it may have influenced treatment response.^42^, ^44^ Although one of the pilot studies included participants with a mean back pain duration of eight years, its small sample size (n = 31) and restriction to only female participants prevent a direct comparison with our study. Another study had 63 participants, women with CLBP randomized to Pilates twice a week for 10 weeks or to a home exercise program. This study demonstrated significant improvement in the well‐validated Roland Morris Disability Questionnaire (RMDQ), but only 3 of 63 participants reported a back pain duration of at least 12 months.^45^

Participants’ duration of back pain in two fully powered efficacy trials was similar to ours, thus permitting more direct comparison of study findings. Morone and colleagues conducted a study of mindfulness meditation in 282 older adults with CLBP and demonstrated significant improvement in pain, but not in the RMDQ.^40^ Karp and colleagues conducted a randomized controlled trial of older adults with comorbid CLBP and depression in which participants were randomized to either pharmacotherapy and supportive therapy or pharmacotherapy and problem‐solving therapy.^46^ Although there was no between‐groups difference in outcomes, both groups improved significantly on the RMDQ.^46^ The findings highlight the importance of treating key nonphysical factors in ameliorating CLBP‐associated disability in older adults and the value of delivering structured, intensive treatment toward demonstrating an impact on outcomes.

Another factor that potentially threatened our ability to demonstrate a significant impact of ABC care on pain‐associated disability was that UC was our control group. Because pain initiatives have been implemented in the VA health care system nationally, usual pain care in the VA is likely distinct from that in general community settings. In 2009 the Veterans Health Administration (VHA) published a directive (2009‐053) that included a stepped‐care model for managing pain in veterans, spanning from foundational services (ie, pain self‐management) to primary care services (step 1), to specialty pain care services (step 2), to tertiary pain care services (step 3). Subsequent research in veterans with chronic pain demonstrated the efficacy of this model in reducing pain‐related disability, pain interference, and pain severity compared with UC as it existed at the time, that is, care not guided by stepped‐care management.^47^ In 2013 the VHA launched another major pain initiative, the Opioid Safety Initiative, that was designed to mitigate high risk prescribing behavior. Because of such initiatives, it is likely that UC in our clinical trial incorporated more robust pain management strategies than that in non‐VA settings.

The impact of ABC care on pain intensity and falls is worth highlighting. Participants in the ABC group reported less present and average pain over the prior week at 6 and 12 months, and marginally fewer falls over the 12 months. The link between pain and falls has been demonstrated by others.^48^ It has been previously demonstrated that a decline in the PROMIS‐Global physical health summary score is associated with increased falls,^49^ and it is noteworthy that our study participants randomized to ABC care had greater improvement in the PROMIS‐Global physical health summary score than those randomized to UC.

The only difference between groups in health care utilization were more office visits in the ABC group than the UC group. This was likely related to the multiple visits associated with many pain interventions in the ABC group, such as acupuncture, chiropractic, physical therapy, and behavioral therapy. It should be highlighted that utilization of health care services was determined by self‐report, which may be less accurate than direct query of electronic medical records, an assessment modality that was not employed in our study. Future studies should consider including more distal measurement of health care utilization (eg, emergency room visits, performance of invasive procedures, opioid prescriptions) as a way to assess an intervention's ultimate impact.

Our findings regarding the prevalence and perceived value of various treatments received are worth highlighting. The prevalence of referral to many pain treatments was greater in the ABC group than the UC group. A key aspect of providing care for patients with chronic pain is active self‐management. It is noteworthy that participants randomized to UC received passive intervention modalities (ie, spinal injections and massage) more often than did those in the ABC group. In addition, it is noteworthy that participants in the UC group rated the perceived value of opioids higher (mean 6.6 on a 10‐point scale) than those randomized to ABC care (mean 5.3 on a 10‐point scale). As we only queried participants about the perceived value of various treatments at the end of the study, we cannot determine the impact of ABC care itself on modifying the perceived value of opioids. It is conceivable that exposure of the ABC participants to more education on the limited role of opioids and the value of nonopioid treatments (per Supplemental Table 5) played a role and should be taken into consideration in future studies.

ABC participants rated the perceived value of two educational components more highly than UC participants—education about causes of their back pain by the doctor (4.9 in ABCs versus 3.5 in UC), and the educational booklet on how to think about pain differently (5.2 in ABCs versus 3.4 in UC). Ample evidence supports the value of education in treating patients with CLBP,^50^ and practitioners should evaluate the quality of educational materials provided before their distribution.

Although our study's sample size, geographic diversity, and incorporation of a comprehensive biopsychosocial approach were strengths, its limitations also should be highlighted. Conducting a study exclusively in veterans naturally limits potential for generalizability to nonveterans. Also, even though ours was the first substantial randomized controlled trial conducted using our algorithms, we used a pragmatic approach. Typically, initial efficacy trials test an intervention that is administered using a rigorous protocol, often overtreating to demonstrate an effect. The nature of our multifaceted intervention, however, did not allow for such an approach. Although beneficial in terms of ease of implementation and application of study findings to the care of veterans in real‐world settings, our intervention's pragmatic nature likely required a significantly larger sample size as well as strategies to promote and measure compliance to demonstrate a significant effect on ODI. As noted above, however, others who have implemented protocolized interventions also have been unable to demonstrate an impact on CLBP‐associated disability.

Given the value of pragmatism as well as protocolization, perhaps future clinical trials might consider implementing elements of both, for example, a structured physical activity program and/or protocolized treatment of key conditions (eg, depression, per the study of Karp et al) in addition to algorithm‐guided care.^46^ Investigators may also wish to consider restricting participants to those with only one or two key disability‐causing conditions. Another limitation of our study is the subjective measure of treatment adherence (ie, provider assessed). A future large study may wish to consider an objective assessment of adherence (eg, attendance of visits to referred providers through collection of electronic medical record data). Additionally, we had a large number of secondary outcomes, and the efficacy with respect to those should be interpreted with caution and as preliminary findings. Given the burgeoning of older adults worldwide, additional research targeting this vulnerable population with CLBP is needed.

## AUTHOR CONTRIBUTIONS

All authors contributed to at least one of the following manuscript preparation roles: conceptualization AND/OR methodology, software, investigation, formal analysis, data curation, visualization, and validation AND drafting or reviewing/editing the final draft. As corresponding author, Dr Fang confirms that all authors have provided the final approval of the version to be published, and takes responsibility for the affirmations regarding article submission (eg, not under consideration by another journal), the integrity of the data presented, and the statements regarding compliance with institutional review board/Declaration of Helsinki requirements.

## Supporting information


**Disclosure Form**:

**Supplemental Table 1.** Reasons for exclusions (N=91).
**Supplemental Table 2:** Continuous non‐primary outcome changes from baseline by intervention group.
**Supplemental Table 3:** Conditions identified at baseline and referrals any time during follow‐up in the ABC group (N=150).
**Supplemental Table 4:** Outcome changes from baseline by intervention group with ABC group restricted to highly compliant cases with participants compliant with treatment for all conditions. Non‐compliance was defined as having a condition and participant refusal of a treatment or having travel or other prohibitive barriers.
**Supplemental Table 5:** Perceived value of received treatment components (0=not helpful; 10=extremely helpful).
