# Supplementary material for: Testing a Personalized Approach to Chronic Low Back Pain: A Randomized Controlled Trial in Older Veterans
Source: Arthritis Care Res (Hoboken). 2026 Feb 8;78(5):609–19. doi: 10.1002/acr.25671 (PMC13116040; doi:10.1002/acr.25671)
Supplement: Supplementary file 2 — Supplemental Table 1. Reasons for exclusions (N=91). Supplemental Table 2: Continuous non‐primary outcome changes from baseline by intervention group. Supplemental Table 3: Conditions identified at baseline and referrals any time during follow‐up in the ABC group (N=150). Supplemental Table 4: Outcome changes from baseline by intervention group with ABC group restricted to highly compliant cases with participants compliant with treatment for all conditions. Non‐compliance was defined as having a condition and participant refusal of a treatment or having travel or other prohibitive barriers. Supplemental Table 5: Perceived value of received treatment components (0=not helpful; 10=extremely helpful). [file ACR-78-609-s001.docx]

Supplemental Table 1: Reasons for exclusions (N=91)

| Reason | N (%) |
| --- | --- |
| Age<65 | 1 (1.1) |
| No LBP | 3 (3.3) |
| Pain duration <6 months | 2 (2.2) |
| Pain not every day | 9 (9.9) |
| No/mild pain only | 4 (4.4) |
| Weight loss | 10 (11.) |
| Fever | 1 (1.1) |
| Worsening pain | 4 (4.4) |
| Lumbar surgery | 13 (14.3) |
| Change in bowel/bladder control | 5 (5.5) |
| Weakness in legs | 10 (11.0) |
| Eye problems | 3 (3.3) |
| Hearing problems | 3 (3.3) |
| Cannot participate 12 months | 13 (14.3) |
| Awakening due to pain | 4 (4.4) |
| Worst pain elsewhere | 4 (4.4) |
| Acute illness | 2 (2.2) |

Supplemental Table 2: Continuous non-primary outcome changes from baseline by intervention group

| Outcome and Period of Change | ABC  (N=150)  Mean±SD | UC  (N=149)  Mean±SD | Adjusted Difference*  Mean±SE | *p*-Value |
| --- | --- | --- | --- | --- |
|  |  |  |  |  |
| PROMIS-29 |  |  |  |  |
| Physical Function |  |  |  |  |
| 3 months | 0.7±5.8 | -0.9±5.5 | 1.0±0.8 | 0.2322 |
| 6 months | 1.0±6.0 | -0.6±5.6 | 1.2±0.7 | 0.0930 |
| 9 months | 1.2±5.7 | 0.6±7.0 | -0.0±0.8 | 0.9589 |
| 12 months | 1.7±6.7 | -0.4±6.1 | 1.9±0.8 | 0.0121 |
|  |  |  |  |  |
| Anxiety |  |  |  |  |
| 3 months | 2.9±8.8 | 2.4±8.7 | 0.4±1.0 | 0.6849 |
| 6 months | 3.0±9.0 | 3.2±8.2 | -0.2±1.0 | 0.8254 |
| 9 months | 2.3±8.8 | 1.2±8.7 | 0.8±1.1 | 0.4780 |
| 12 months | 2.4±8.3 | 3.2±10.0 | -0.5±1.1 | 0.6477 |
|  |  |  |  |  |
| Depression |  |  |  |  |
| 3 months | 1.9±7.5 | 3.2±7.4 | -0.3±1.0 | 0.7793 |
| 6 months | 1.4±7.8 | 3.4±6.8 | -1.3±0.8 | 0.1179 |
| 9 months | 1.4±8.2 | 2.4±7.2 | -0.5±1.0 | 0.6405 |
| 12 months | 2.0±8.6 | 3.4±8.2 | -1.2±1.0 | 0.2052 |
|  |  |  |  |  |
| Fatigue |  |  |  |  |
| 3 months | -0.4±9.8 | 2.5±8.4 | -1.9±1.0 | 0.0593 |
| 6 months | 0.4±9.5 | 2.0±9.4 | -1.0±1.0 | 0.3185 |
| 9 months | 0.1±10.1 | 1.3±9.7 | -0.9±1.1 | 0.4293 |
| 12 months | -1.2±9.9 | 1.4±8.9 | -1.8±1.1 | 0.0824 |
|  |  |  |  |  |
| Sleep Disturbance |  |  |  |  |
| 3 months | 0.0±7.9 | -1.6±7.6 | 0.6±0.9 | 0.5287 |
| 6 months | -0.5±9.0 | -1.6±7.9 | -0.0±0.9 | 0.9717 |
| 9 months | -0.2±8.0 | -1.8±7.4 | 0.1±1.0 | 0.9523 |
| 12 months | -0.7±9.1 | -1.4±8.1 | -0.6±1.0 | 0.5587 |
|  |  |  |  |  |
| Social Roles |  |  |  |  |
| 3 months | 0.2±8.4 | -0.8±9.1 | 0.6±1.0 | 0.5690 |
| 6 months | 0.4±8.1 | -0.2±9.8 | 0.5±1.0 | 0.5942 |
| 9 months | 0.1±9.2 | 0.7±9.9 | 0.8±1.1 | 0.4906 |
| 12 months | -0.6±9.4 | 0.6±10.2 | -0.2±1.1 | 0.8211 |
|  |  |  |  |  |
| Pain Interference |  |  |  |  |
| 3 months | -0.4±6.9 | -0.6±7.1 | -0.1±0.8 | 0.8932 |
| 6 months | -1.3±6.6 | -1.0±8.2 | -0.2±0.8 | 0.8244 |
| 9 months | -1.8±7.6 | -0.3±7.5 | -1.2±1.0 | 0.2032 |
| 12 months | -0.4±6.9 | -0.8±8.5 | -0.0±0.9 | 0.9736 |
|  |  |  |  |  |
| Average Pain |  |  |  |  |
| 3 months | -0.5±2.1 | -0.4±1.9 | -0.2±0.2 | 0.3568 |
| 6 months | -0.8±2.1 | -0.5±2.2 | -0.5±0.3 | 0.0589 |
| 9 months | -1.2±2.3 | -0.6±2.0 | -0.5±0.3 | 0.0779 |
| 12 months | -0.9±2.4 | -0.5±2.0 | -0.4±0.3 | 0.1189 |
|  |  |  |  |  |
| PROMIS Global Health |  |  |  |  |
| General Health |  |  |  |  |
| 3 months | -0.2±0.7 | -0.1±0.7 | -0.0±0.1 | 0.7257 |
| 6 months | -0.2±0.7 | -0.2±0.8 | 0.1±0.1 | 0.5390 |
| 9 months | -0.1±0.7 | -0.2±0.8 | 0.1±0.1 | 0.1687 |
| 12 months | -0.2±0.7 | -0.4±0.8 | 0.2±0.1 | 0.0404 |
|  |  |  |  |  |
| Roles |  |  |  |  |
| 3 months | -0.1±1.0 | -0.2±1.1 | 0.1±0.1 | 0.6523 |
| 6 months | -0.1±0.9 | -0.2±1.1 | 0.0±0.1 | 0.7101 |
| 9 months | -0.3±1.0 | -0.4±1.2 | -0.0±0.1 | 0.9982 |
| 12 months | -0.2±1.0 | -0.4±1.2 | 0.1±0.1 | 0.4929 |
|  |  |  |  |  |
| Physical Health |  |  |  |  |
| 3 months | 0.7±5.7 | -0.6±5.3 | 1.2±0.8 | 0.1218 |
| 6 months | 1.3±6.3 | -1.2±5.9 | 2.4±0.7 | 0.0008 |
| 9 months | 0.9±6.3 | -1.3±5.4 | 2.1±0.8 | 0.0088 |
| 12 months | 0.7±6.1 | -1.5±6.1 | 2.0±0.8 | 0.0118 |
|  |  |  |  |  |
| Mental Health |  |  |  |  |
| 3 months | -0.2±6.3 | -1.5±6.1 | 1.0±0.9 | 0.2730 |
| 6 months | 0.2±6.4 | -2.3±6.2 | 1.9±0.7 | 0.0100 |
| 9 months | -0.5±6.8 | -1.7±6.3 | 1.0±0.9 | 0.2354 |
| 12 months | -1.8±7.0 | -2.8±6.9 | 0.8±0.8 | 0.3481 |
|  |  |  |  |  |
| Falls Efficacy Scale |  |  |  |  |
| 3 months | 0.9±4.3 | 1.9±4.3 | -1.1±0.5 | 0.0446 |
| 6 months | 0.5±4.8 | 1.6±4.4 | -0.9±0.6 | 0.1182 |
| 9 months | 1.3±5.2 | 1.2±5.0 | 0.2±0.6 | 0.7164 |
| 12 months | 1.3±4.6 | 1.6±5.1 | -0.1±0.6 | 0.8655 |
|  |  |  |  |  |
| Life Space Assessment |  |  |  |  |
| 3 months | -2.8±22.3 | -3.0±24.5 | 0.0±2.6 | 0.9880 |
| 6 months | -2.0±22.3 | -5.7±24.9 | 3.2±2.4 | 0.1839 |
| 9 months | -7.2±25.1 | -5.9±22.8 | -3.1±2.9 | 0.2774 |
| 12 months | -4.0±24.4 | -6.3±26.4 | 0.5±2.7 | 0.8595 |
|  |  |  |  |  |
| VR-12 |  |  |  |  |
| Physical Summary |  |  |  |  |
| 3 months | 1.1±8.4 | -1.2±9.1 | 2.2±1.0 | 0.0415 |
| 6 months | 1.3±8.4 | -1.1±8.6 | 2.2±1.1 | 0.0553 |
| 9 months | 0.8±9.1 | -0.5±7.8 | 1.4±1.1 | 0.2314 |
| 12 months | -0.0±9.6 | -0.9±8.5 | 0.8±1.1 | 0.4594 |
|  |  |  |  |  |
| Mental Summary |  |  |  |  |
| 3 months | -0.7±10.2 | -0.4±9.4 | -0.5±1.2 | 0.6791 |
| 6 months | 1.5±10.7 | -0.8±9.3 | 1.7±1.2 | 0.1624 |
| 9 months | 0.8±11.3 | -0.2±9.7 | 0.6±1.3 | 0.6602 |
| 12 months | -0.2±11.5 | -1.6±10.1 | 0.7±1.3 | 0.5880 |
|  |  |  |  |  |
| Pain Scale |  |  |  |  |
| Pain at the Moment |  |  |  |  |
| 1 month | 0.4±2.4 | 0.4±2.1 | -0.3±0.3 | 0.3820 |
| 2 months | 0.3±2.5 | 0.6±2.6 | -0.4±0.3 | 0.1295 |
| 3 months | -0.3±2.5 | 0.5±2.2 | -0.6±0.3 | 0.0164 |
| 4 months | -0.2±2.2 | 0.1±2.3 | -0.5±0.3 | 0.0588 |
| 5 months | -0.1±2.7 | 0.5±2.5 | -0.7±0.3 | 0.0121 |
| 6 months | -0.5±2.4 | 0.0±2.4 | -0.6±0.3 | 0.0348 |
| 7 months | -0.2±2.6 | -0.1±2.8 | -0.2±0.3 | 0.5480 |
| 8 months | -0.4±2.4 | -0.2±2.8 | -0.2±0.3 | 0.4598 |
| 9 months | -0.4±2.8 | 0.2±2.2 | -0.7±0.3 | 0.0334 |
| 10 months | -0.7±2.7 | -0.0±2.2 | -0.6±0.3 | 0.0465 |
| 11 months | 0.0±3.0 | 0.2±2.4 | -0.3±0.3 | 0.3316 |
| 12 months | -0.3±2.6 | 0.3±2.6 | -0.7±0.3 | 0.0198 |
|  |  |  |  |  |
| Average Pain Prior Week |  |  |  |  |
| 1 month | -0.3±1.7 | -0.1±2.0 | -0.4±0.2 | 0.1405 |
| 2 months | -0.0±1.8 | -0.2±2.0 | -0.0±0.3 | 0.8768 |
| 3 months | -0.6±2.1 | -0.5±2.1 | -0.2±0.2 | 0.4447 |
| 4 months | -0.8±1.9 | -0.4±2.1 | -0.5±0.3 | 0.0461 |
| 5 months | -0.7±2.0 | -0.5±1.8 | -0.3±0.2 | 0.2123 |
| 6 months | -0.8±2.1 | -0.4±2.1 | -0.5±0.2 | 0.0412 |
| 7 months | -0.8±2.2 | -0.6±2.5 | -0.3±0.3 | 0.3075 |
| 8 months | -0.9±2.2 | -0.6±2.1 | -0.3±0.3 | 0.2604 |
| 9 months | -1.0±2.1 | -0.3±1.9 | -0.6±0.3 | 0.0273 |
| 10 months | -1.0±2.1 | -0.4±2.1 | -0.6±0.3 | 0.0455 |
| 11 months | -0.5±2.3 | -0.5±1.8 | -0.0±0.3 | 0.9342 |
| 12 months | -0.9±2.4 | -0.3±2.1 | -0.6±0.3 | 0.0220 |
|  |  |  |  |  |
| Worst Pain Prior Week |  |  |  |  |
| 1 month | -0.3±1.8 | -0.4±2.2 | -0.0±0.3 | 0.8703 |
| 2 months | -0.2±2.0 | -0.4±2.1 | 0.0±0.3 | 0.9521 |
| 3 months | -0.7±2.5 | -0.8±2.3 | -0.1±0.3 | 0.8302 |
| 4 months | -0.8±2.2 | -0.7±2.2 | -0.3±0.3 | 0.1896 |
| 5 months | -1.0±2.3 | -0.7±2.0 | -0.3±0.3 | 0.2528 |
| 6 months | -1.0±2.6 | -0.7±2.4 | -0.4±0.3 | 0.1286 |
| 7 months | -0.9±2.7 | -0.8±2.7 | -0.2±0.3 | 0.5744 |
| 8 months | -1.3±2.6 | -0.7±2.4 | -0.6±0.3 | 0.0632 |
| 9 months | -1.2±2.6 | -0.4±2.3 | -0.7±0.3 | 0.0220 |
| 10 months | -1.4±2.5 | -0.6±1.9 | -0.7±0.3 | 0.0242 |
| 11 months | -0.9±2.5 | -0.5±1.9 | -0.2±0.3 | 0.6113 |
| 12 months | -1.3±2.9 | -0.6±2.3 | -0.6±0.3 | 0.0334 |

SD=Standard deviation

SE=Standard error

* Adjusted for pre-administration value of the physiologic measure as a covariate using linear mixed models and multiple imputation for missing data

Supplemental Table 3: Conditions identified at baseline and referrals any time during follow-up in the ABC group (N=150)

| Condition and Referral | N (%) |
| --- | --- |
| Anxiety | 31 (20.7) |
| Referrals to behavioral health | 8 (25.8) |
|  |  |
| Depression | 36 (24.0) |
| Referrals to behavioral health | 11 (30.6) |
|  |  |
| Fibromyalgia | 4 (2.7) |
| Referrals to behavioral health | 2 (50.0) |
| Referrals to interdisciplinary rehabilitation | 1 (25.0) |
|  |  |
| Hip osteoarthritis | 33 (22.0) |
| Referrals to hip injection | 9 (27.3) |
| Referrals to physical therapy | 6 (18.2) |
| Referrals to orthopedics | 3 (9.1) |
| Referrals to acupuncture | 8 (24.2) |
| Referrals to interdisciplinary rehabilitation | 1 (3.0) |
|  |  |
| Insomnia | 68 (54.3) |
| Referrals to non-pharmacological intervention | 21 (30.9) |
|  |  |
| Lateral hip/thigh pain | 38 (25.3) |
| Referrals to physical therapy | 8 (21.1) |
|  |  |
| Leg length discrepancy | 17 (11.3) |
| Referrals to physical therapy | 5 (29.4) |
|  |  |
| Lumbar spinal stenosis | 45 (30.0) |
| Referrals to physical therapy | 7 (15.6) |
| Referrals to epidural injection | 5 (11.1) |
| Referrals to acupuncture | 13 (28.9) |
| Referrals to surgical evaluation | 4 (8.9) |
|  |  |
| Maladaptive coping | 43 (28.7) |
| Referrals to behavioral health | 14 (32.6) |
| Referrals to physical therapy | 11 (25.6) |
|  |  |
| Myofascial pain | 119 (79.3) |
| Referrals to physical therapy | 40 (33.6) |
| Referrals to chiropractic | 25 (21.0) |
| Referrals to acupuncture | 47 (39.5) |
| Referrals to trigger point injection | 4 (3.4) |
| Referrals to interdisciplinary rehabilitation | 17 (14.3) |
|  |  |
| SI joint syndrome | 51 (34.0) |
| Referrals to physical therapy | 8 (15.7) |
| Referrals to chiropractic | 8 (15.7) |
| Referrals to acupuncture | 18 (35.3) |
| Referrals to joint injection | 3 (5.9) |
|  |  |
| One or more CNS condition/s (anxiety, depression, fibromyalgia, insomnia, maladaptive coping) | 101 (67.3) |
|  |  |
| One or more physical condition/s (hip OA, lateral hip/thigh pain, leg length discrepancy, lumbar spinal stenosis, myofascial pain, SI joint syndrome) | 135 (90.0) |
|  |  |
| Both CNS and physical conditions | 97 (64.7) |

Supplemental Table 4: Outcome changes from baseline by intervention group with ABC group restricted to highly compliant cases with participants compliant with treatment for all conditions. Non-compliance was defined as having a condition and participant refusal of a treatment or having travel or other prohibitive barriers.

| Outcome and Period of Change | ABC  (N=106)  Mean±SD | UC  (N=149)  Mean±SD | Adjusted Difference*  Mean±SE | *p*-Value |
| --- | --- | --- | --- | --- |
| Oswestry Disability Index |  |  |  |  |
| 3 months | -0.8±13.4 | 2.7±11.3 | -3.2±1.8 | 0.0776 |
| 6 months | -2.9±11.4 | 1.2±11.5 | -3.1±1.7 | 0.0612 |
| 9 months | -0.4±13.5 | -0.2±12.6 | -1.8±1.9 | 0.3333 |
| 12 months | -0.5±13.8 | 2.8±12.1 | -3.2±1.8 | 0.0786 |
|  |  |  |  |  |
| PROMIS-29 |  |  |  |  |
| Physical Function |  |  |  |  |
| 3 months | 0.1±5.3 | -0.9±5.5 | 0.8±0.8 | 0.3180 |
| 6 months | 0.8±6.0 | -0.6±5.6 | 1.3±0.8 | 0.0802 |
| 9 months | 0.2±4.7 | 0.6±7.0 | -0.3±0.8 | 0.7065 |
| 12 months | 1.4±6.6 | -0.4±6.1 | 1.8±0.8 | 0.0213 |
|  |  |  |  |  |
| Anxiety |  |  |  |  |
| 3 months | 2.6±8.9 | 2.4±8.7 | 0.1±1.1 | 0.9070 |
| 6 months | 2.0±7.9 | 3.2±8.2 | -0.8±1.1 | 0.4677 |
| 9 months | 1.0±7.9 | 1.2±8.7 | -0.3±1.3 | 0.8362 |
| 12 months | 2.0±8.6 | 3.2±10.0 | -0.6±1.2 | 0.6010 |
|  |  |  |  |  |
| Depression |  |  |  |  |
| 3 months | 1.9±7.1 | 3.2±7.4 | -0.4±1.0 | 0.7058 |
| 6 months | 0.9±7.4 | 3.4±6.8 | -1.4±1.0 | 0.1509 |
| 9 months | 0.3±7.4 | 2.4±7.2 | -1.1±1.1 | 0.3105 |
| 12 months | 1.6±8.2 | 3.4±8.2 | -1.5±1.1 | 0.1585 |
|  |  |  |  |  |
| Fatigue |  |  |  |  |
| 3 months | 1.0±9.1 | 2.5±8.4 | -1.4±1.0 | 0.1804 |
| 6 months | 0.5±9.4 | 2.0±9.4 | -0.9±1.1 | 0.4079 |
| 9 months | 0.3±9.3 | 1.3±9.7 | -1.3±1.3 | 0.3059 |
| 12 months | -0.5±9.6 | 1.4±8.9 | -1.7±1.2 | 0.1543 |
|  |  |  |  |  |
| Sleep Disturbance |  |  |  |  |
| 3 months | 0.2±8.1 | -1.6±7.6 | 0.8±0.9 | 0.3735 |
| 6 months | -0.7±9.1 | -1.6±7.9 | -0.1±0.9 | 0.9400 |
| 9 months | -0.4±8.0 | -1.8±7.4 | 0.3±1.1 | 0.7963 |
| 12 months | -1.2±9.3 | -1.4±8.1 | -0.6±1.1 | 0.6102 |
|  |  |  |  |  |
| Social Roles |  |  |  |  |
| 3 months | 0.8±8.6 | -0.8±9.1 | 1.3±1.1 | 0.2490 |
| 6 months | 1.6±8.1 | -0.2±9.8 | 1.0±1.1 | 0.3484 |
| 9 months | 1.5±8.0 | 0.7±9.9 | 1.6±1.2 | 0.1826 |
| 12 months | 0.9±8.7 | 0.6±10.2 | 0.7±1.2 | 0.5630 |
|  |  |  |  |  |
| Pain Interference |  |  |  |  |
| 3 months | -0.6±6.9 | -0.6±7.1 | -0.4±0.9 | 0.6788 |
| 6 months | -2.2±5.8 | -1.0±8.2 | -0.7±0.9 | 0.4782 |
| 9 months | -2.1±7.2 | -0.3±7.5 | -1.5±1.0 | 0.1595 |
| 12 months | -0.7±6.3 | -0.8±8.5 | -0.0±1.0 | 0.9860 |
|  |  |  |  |  |
| Average Pain |  |  |  |  |
| 3 months | -0.5±2.1 | -0.4±1.9 | -0.2±0.3 | 0.4293 |
| 6 months | -0.9±2.1 | -0.5±2.2 | -0.5±0.3 | 0.0551 |
| 9 months | -1.1±2.0 | -0.6±2.0 | -0.5±0.3 | 0.0803 |
| 12 months | -1.1±2.4 | -0.5±2.0 | -0.7±0.3 | 0.0199 |
|  |  |  |  |  |
| PROMIS Global Health |  |  |  |  |
| General Health |  |  |  |  |
| 3 months | -0.3±0.8 | -0.1±0.7 | -0.1±0.1 | 0.2519 |
| 6 months | -0.1±0.6 | -0.2±0.8 | 0.0±0.1 | 0.6451 |
| 9 months | -0.2±0.7 | -0.2±0.8 | 0.2±0.1 | 0.1230 |
| 12 months | -0.2±0.7 | -0.4±0.8 | 0.2±0.1 | 0.1559 |
|  |  |  |  |  |
| Roles |  |  |  |  |
| 3 months | -0.2±1.0 | -0.2±1.1 | 0.1±0.1 | 0.3302 |
| 6 months | -0.1±0.8 | -0.2±1.1 | 0.1±0.1 | 0.5335 |
| 9 months | -0.3±1.0 | -0.4±1.2 | 0.0±0.1 | 0.9633 |
| 12 months | -0.2±1.0 | -0.4±1.2 | 0.1±0.1 | 0.4925 |
|  |  |  |  |  |
| Physical Function |  |  |  |  |
| 3 months | 0.9±5.6 | -0.6±5.3 | 1.5±0.7 | 0.0352 |
| 6 months | 2.1±5.8 | -1.2±5.9 | 2.5±0.7 | 0.0007 |
| 9 months | 1.4±6.3 | -1.3±5.4 | 2.7±0.9 | 0.0026 |
| 12 months | 1.2±5.7 | -1.5±6.1 | 2.3±0.8 | 0.0060 |
|  |  |  |  |  |
| Mental Function |  |  |  |  |
| 3 months | -0.2±6.7 | -1.5±6.1 | 1.1±0.8 | 0.1938 |
| 6 months | 0.4±6.6 | -2.3±6.2 | 1.8±0.8 | 0.0285 |
| 9 months | -0.1±6.5 | -1.7±6.3 | 1.5±1.0 | 0.1220 |
| 12 months | -1.9±7.2 | -2.8±6.9 | 0.8±0.9 | 0.3826 |
|  |  |  |  |  |
| Falls Efficacy Scale |  |  |  |  |
| 3 months | 0.8±4.2 | 1.9±4.3 | -1.2±0.6 | 0.0716 |
| 6 months | 0.3±4.7 | 1.6±4.4 | -1.1±0.6 | 0.0613 |
| 9 months | 1.1±4.7 | 1.2±5.0 | -0.1±0.7 | 0.8563 |
| 12 months | 1.0±4.4 | 1.6±5.1 | -0.5±0.7 | 0.4202 |
|  |  |  |  |  |
| Life Space Assessment |  |  |  |  |
| 3 months | -4.1±22.3 | -3.0±24.5 | 0.6±2.8 | 0.8212 |
| 6 months | -2.6±21.9 | -5.7±24.9 | 3.2±2.8 | 0.2530 |
| 9 months | -9.3±24.9 | -5.9±22.8 | -3.3±3.2 | 0.3039 |
| 12 months | -4.0±23.9 | -6.3±26.4 | -2.0±3.1 | 0.5086 |
|  |  |  |  |  |
| VR-12 |  |  |  |  |
| Physical Summary |  |  |  |  |
| 3 months | 0.4±8.1 | -0.9±9.2 | 1.6±1.2 | 0.1908 |
| 6 months | 2.1±8.3 | -0.8±8.8 | 2.7±1.1 | 0.0170 |
| 9 months | 1.8±8.7 | -0.3±7.9 | 2.4±1.3 | 0.0648 |
| 12 months | 0.9±9.3 | -0.8±8.7 | 1.4±1.2 | 0.2688 |
|  |  |  |  |  |
| Mental Summary |  |  |  |  |
| 3 months | 0.0±9.1 | -0.7±9.2 | 0.2±1.3 | 0.8638 |
| 6 months | 1.6±10.0 | -1.0±9.0 | 1.6±1.2 | 0.1891 |
| 9 months | 1.2±10.7 | -0.5±9.4 | 1.2±1.5 | 0.4285 |
| 12 months | -0.3±11.0 | -1.9±9.9 | 0.8±1.4 | 0.5645 |
|  |  |  |  |  |
| Pain Scale |  |  |  |  |
| Pain at the Moment |  |  |  |  |
| 1 month | 0.1±2.4 | 0.4±2.1 | -0.6±0.3 | 0.0312 |
| 2 months | 0.1±2.6 | 0.6±2.6 | -0.5±0.3 | 0.0748 |
| 3 months | -0.3±2.4 | 0.5±2.2 | -0.7±0.3 | 0.0166 |
| 4 months | -0.1±2.1 | 0.1±2.3 | -0.5±0.3 | 0.0962 |
| 5 months | 0.0±2.5 | 0.5±2.5 | -0.7±0.3 | 0.0317 |
| 6 months | -0.7±2.5 | 0.0±2.4 | -0.6±0.3 | 0.0305 |
| 7 months | -0.5±2.5 | -0.1±2.8 | -0.4±0.3 | 0.3015 |
| 8 months | -0.6±2.4 | -0.2±2.8 | -0.3±0.3 | 0.4403 |
| 9 months | -0.6±2.8 | 0.2±2.2 | -0.7±0.3 | 0.0264 |
| 10 months | -0.9±2.8 | -0.0±2.2 | -0.7±0.4 | 0.0347 |
| 11 months | -0.6±2.9 | 0.2±2.4 | -0.6±0.4 | 0.0932 |
| 12 months | -0.7±2.6 | 0.3±2.6 | -0.9±0.3 | 0.0049 |
|  |  |  |  |  |
| Average Pain Prior Week |  |  |  |  |
| 1 month | -0.3±1.7 | -0.1±2.0 | -0.4±0.3 | 0.1039 |
| 2 months | -0.1±1.9 | -0.2±2.0 | -0.1±0.3 | 0.7507 |
| 3 months | -0.7±2.2 | -0.5±2.1 | -0.3±0.3 | 0.2622 |
| 4 months | -0.8±1.9 | -0.4±2.1 | -0.5±0.2 | 0.0383 |
| 5 months | -0.7±2.0 | -0.5±1.8 | -0.3±0.3 | 0.2495 |
| 6 months | -1.0±2.2 | -0.4±2.1 | -0.6±0.3 | 0.0212 |
| 7 months | -1.1±2.2 | -0.6±2.5 | -0.7±0.3 | 0.0312 |
| 8 months | -0.9±2.2 | -0.6±2.1 | -0.3±0.3 | 0.3457 |
| 9 months | -1.1±2.2 | -0.3±1.9 | -0.8±0.3 | 0.0082 |
| 10 months | -0.9±2.0 | -0.4±2.1 | -0.6±0.3 | .0446 |
| 11 months | -0.7±2.5 | -0.5±1.8 | -0.2±0.3 | 0.4848 |
| 12 months | -1.0±2.4 | -0.3±2.1 | -0.8±0.3 | 0.0093 |
|  |  |  |  |  |
| Worst Pain Prior Week |  |  |  |  |
| 1 month | -0.5±1.8 | -0.4±2.2 | -0.2±0.3 | 0.5868 |
| 2 months | -0.3±2.1 | -0.4±2.1 | -0.0±0.3 | 0.9672 |
| 3 months | -0.6±2.4 | -0.8±2.3 | -0.1±0.3 | 0.6788 |
| 4 months | -0.8±2.1 | -0.7±2.2 | -0.2±0.3 | 0.3936 |
| 5 months | -1.2±2.1 | -0.7±2.0 | -0.4±0.3 | 0.1244 |
| 6 months | -1.4±2.6 | -0.7±2.4 | -0.7±0.3 | 0.0166 |
| 7 months | -1.1±2.7 | -0.8±2.7 | -0.4±0.3 | 0.2030 |
| 8 months | -1.4±2.5 | -0.7±2.4 | -0.6±0.3 | 0.0555 |
| 9 months | -1.5±2.7 | -0.4±2.3 | -0.9±0.3 | 0.0059 |
| 10 months | -1.5±2.2 | -0.6±1.9 | -0.8±0.3 | 0.0143 |
| 11 months | -1.2±2.5 | -0.5±1.9 | -0.4±0.3 | 0.2825 |
| 12 months | -1.5±3.0 | -0.6±2.3 | -0.8±0.3 | 0.0158 |

SD=Standard deviation

SE=Standard error

* Adjusted for pre-administration value of the physiologic measure as a covariate using linear mixed models and multiple imputation for missing data

Supplemental Table 5: Perceived value of received treatment components (0=not helpful; 10=extremely helpful)

| Treatment Component | ABC  (N=150)  [Number Received]  Mean Rating | UC  (N=149)  [Number Received]  Mean Rating |
| --- | --- | --- |
| Physical therapy | [78] 5.9 | [35] 5.4 |
| Chiropractic | [53] 6.6 | [27] 5.6 |
| Acupuncture | [66] 6.3 | [20] 4.4 |
| Opioid pain medication | [29] 5.3 | [26] 6.6 |
| Non-opioid pain medication | [96] 5.8 | [82] 6.0 |
| Home exercise program | [95] 6.8 | [74] 6.2 |
| Monthly telephone calls by research coordinator | [107] 5.3 | [102] 5.8 |
| Education about the causes of your back pain by the doctor | [55] 4.9 | [32] 3.5 |
| Educational booklet on how to think about your pain differently | [56] 5.2 | [29] 3.4 |
| Weight loss | [49] 5.5 | [36] 5.7 |
| Aquatherapy | [21] 6.3 | [12] 6.9 |
| Shots in spine | [13] 5.5 | [21] 5.1 |
| Shots in muscles | [12] 5.5 | [13] 5.3 |
| Learning how to manage pain on own | [59] 6.6 | [55] 6.5 |
| Cognitive behavioral therapy by a psychologist | [18] 6.4 | [8] 5.8 |
| Massage | [15] 7.4 | [21] 6.8 |
| Yoga | [11] 4.5 | [8] 6.8 |
| T’ai Chi | [13] 4.7 | 1 [5.0] |
